# Sleep monitoring by actigraphy in short-stay ICU patients

**DOI:** 10.1186/cc10927

**Published:** 2012-03-20

**Authors:** AW Van der Kooi, JH Tulen, AW De Weerd, MM Van Eijk, MJ Van Uitert, SE De Rooij, BC Van Munster, AJ Slooter

**Affiliations:** 1 University Medical Center Utrecht, the Netherlands; 2 Erasmus MC, University Medical Center Rotterdam, the Netherlands; 3 Sein, Zwolle, the Netherlands; 4 Academic Medical Center, Amsterdam, the Netherlands

## Introduction

Sleep deprivation is common in ICU patients, but difficult to investigate [1]. The gold standard for sleep monitoring, polysomnography (PSG), is impractical for use in ICU patients [2]. Actigraphy proved to be a good alternative in non-ICU patients [3]. However, in prolonged mechanically ventilated patients, actigraphy was inaccurate, probably due to ICU-acquired weakness and resulting inactivity [2]. Short-stay ICU patients do not suffer from ICU-acquired weakness, and the accuracy of actigraphy in these patients has not yet been studied [4]. The aim of this study was to investigate actigraphy for sleep assessment in short-stay ICU patients.

## Methods

PSG and actigraphy measurements were conducted in seven postcardiothoracic surgery patients. Total sleep time, sleep efficiency, number of awakenings and wake time after sleep onset were determined with actigraphy and compared to PSG. The accuracy, sensitivity (percentage correctly scored as sleep) and specificity (percentage correctly scored as awake) were calculated for actigraphy using high, medium, low and automatic threshold sensitivity settings of the actigraphy software.

## Results

The only parameter that showed a significant correlation between PSG and actigraphy was the number of awakenings (*r *= 0.76, *P *= 0.049, high threshold setting). Actigraphy underestimated wake time after sleep onset and overestimated total sleep time and sleep efficiency. The median specificity for actigraphy was below 19% and the median sensitivity above 94% for all threshold settings.

## Conclusion

Actigraphy is not reliable for one-night sleep-wake detection in short-stay postoperative ICU patients.
